# Pericardial Closure Using Donor Pericardium in Deceased-Donor Lung Transplantation

**DOI:** 10.5761/atcs.oa.26-00023

**Published:** 2026-04-22

**Authors:** Taiki Ryo, Akihiro Ohsumi, Ichiro Sakanoue, Hidenao Kayawake, Satona Tanaka, Yojiro Yutaka, Daisuke Nakajima, Hiroshi Date

**Affiliations:** 1 Department of Thoracic Surgery, Kyoto University, Kyoto, Kyoto, Japan; 2 Department of Thoracic Surgery, Nagoya University, Nagoya, Aichi, Japan; 3 Department of Thoracic Surgery, Tazuke Kofukai Medical Research Institute, Kitano Hospital, Osaka, Osaka, Japan; 4 Department of Surgery, Duke University Medical Center, Durham, NC, USA

**Keywords:** donor pericardium, lung transplantation, adhesion

## Abstract

**Purpose:**

The optimal method of pericardial closure in lung transplantation remains controversial. This study aimed to evaluate the technical feasibility and procedural simplicity of pericardial closure using donor pericardium.

**Methods:**

We retrospectively reviewed 70 adult patients who underwent deceased-donor bilateral lung transplantation with cardiopulmonary bypass or central extracorporeal membrane oxygenation between December 2010 and January 2024 at Kyoto University Hospital. Patients were divided into the Donor pericardium group (n = 21) and the Autologous tissue group (n = 49). Pericardial closure time was assessed as an indicator of procedural simplicity, and postoperative complications within 1 year were descriptively evaluated.

**Results:**

Baseline characteristics did not differ between the groups. Mean pericardial closure time was significantly shorter in the Donor pericardium group than in the Autologous tissue group (13 ± 2.4 vs 19 ± 1.6 min, P = 0.03). No clinically concerning differences in postoperative complications were observed during short- to mid-term follow-up.

**Conclusion:**

Pericardial closure using donor pericardium enables technically simpler and faster closure without apparent short-term safety concerns.

## Introduction

In lung transplantation, consensus regarding the necessity or optimal technique of pericardial closure is lacking. At our institution, pericardial closure has been performed to reduce adhesions between the sternum and heart, particularly in the setting of potential lung retransplantation. In cardiac surgery, reconstruction of the pericardium using synthetic substitutes has been reported to reduce cardiac injury during resternotomy by acting as a barrier between the sternum and heart.^1)^ This concept provides a theoretical rationale for pericardial reconstruction when future reentry may be anticipated. In addition, in the context of transplantation, where long-term immunosuppression is required, the routine use of artificial materials may be undesirable, and reconstruction using autologous or biologic tissues may therefore be preferable when feasible. Initially, autologous tissues, including the thymus or pericardial fat, were used to fill the space between the opened pericardium; however, this approach was often time-consuming, especially in patients with cardiac enlargement due to pulmonary hypertension.

For procedural simplicity and adequate coverage, donor pericardium has been adopted since 2020. In deceased-donor lung transplantation (DDLT), donor pericardium has previously been used to correct discrepancies in pulmonary artery or left atrial cuff dimensions.^2,3)^ However, the technical feasibility of using donor pericardium for closure of the recipient’s pericardial sac has not been well described. This study evaluated the feasibility and procedural characteristics of pericardial closure using donor pericardium.

## Materials and Methods

### Patient selection

This retrospective cohort study was conducted in accordance with the ethical standards of the responsible committee on human experimentation (institutional and national) and the 1964 Declaration of Helsinki and its subsequent revisions. This study was approved by the Kyoto University Certified Review Board, and written informed consent was obtained from the patients (approval number: R2389-6; approval date: March 25, 2025).

**Fig. 1:**
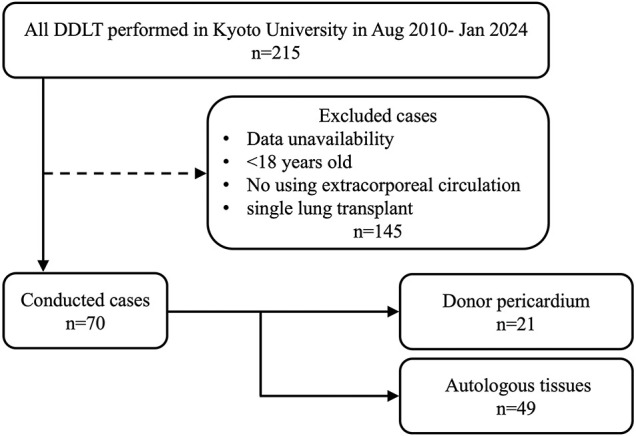
Flowchart of patient selection.

A total of 215 patients underwent DDLT between August 2010 and January 2024 at Kyoto University Hospital. Among them, 145 were excluded, including those without surgical videos, those <18 years old, those who did not undergo extracorporeal circulation, and those who underwent single lung transplant, where the pericardium was not opened or had a minimal incision that could be directly closed. Ultimately, 70 patients with wide pericardial incisions were included (**Fig. 1**). Autologous tissue was used from December 2010 to September 2020, whereas donor pericardium was used from October 2020 to January 2024.

### Pericardial closure techniques (Video 1)

#### Autologous tissue

The thymus and pericardial fat were trimmed intraoperatively and used to fill the space between the opened pericardial edges.

#### Donor pericardium

**Fig. 2:**
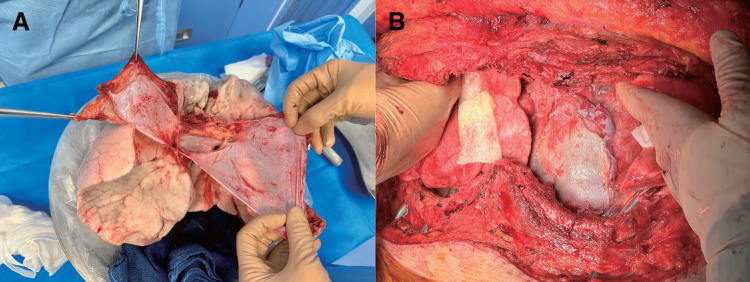
The pericardium was secured from a deceased donor during lung procurement (**A**). Donor pericardium at delayed chest closure on postoperative day 5 (**B**).

During donor lung procurement, the pericardium was preserved (**Fig. 2A**). After removal of attached fat tissue, the donor pericardium was sutured to cover the pericardial defect.

### Outcome assessment

Pericardial closure time was evaluated as an indicator of procedural simplicity and technical feasibility.

Postoperative complications within 1 year were compared between the Autologous tissue group and the Donor pericardium group. Additionally, the effect of donor pericardium on acute pulmonary rejection was assessed. The occurrence of heart failure and pericardial effusion within 1 year postoperatively was evaluated by scheduled transthoracic echocardiography at 1, 3, 6, and 12 months after transplantation. In cases in which constrictive pericarditis was suspected on transthoracic echocardiography, the diagnosis was confirmed by right heart catheterization. In the Donor pericardium group, potential indirect evidence of donor pericardium rejection was assessed by the presence of pericardial effusion or thickening on computed tomography performed during episodes of acute lung rejection.

### Statistical analysis

Clinical information, including procedures and complications, was examined by chart review. Continuous variables were presented as interquartile ranges. Categorical variables were summarized using frequency distribution tables. Between-group comparisons of continuous variables were performed using the Mann–Whitney U test. Categorical variables were compared using the chi-squared test; however, Fisher’s exact test was applied when expected frequencies in any cell were below 5. All statistical analyses were conducted using JMP software, version 18.0.2 (SAS Institute, Cary, NC, USA).

## Results

Donor pericardium was used in 21 cases, whereas autologous tissues were used in 49. **Table 1** summarizes the patient characteristics. There were no differences between the 2 groups in terms of age, gender, underlying diseases,or the history of prior thoracic surgery.

**Table 1:** Patients’ characteristics

	Autologous (n = 49)	Donor (n = 21)	*P*-value
Age, mean (range)	41 (19–56)	38 (19–58)	0.38
Sex (%)			0.79
Male	19 (38.8)	9 (42.9)	
Female	30 (61.2)	12 (57.1)	
Disease (%)			0.16
IPF	15 (30.6)	3 (14.3)	
IPAH	17 (34.7)	4 (19.0)	
Lung injury after HSCT	6 (12.2)	2 (9.5)	
Others	11 (22.4)	12 (57.1)	
History of prior thoracic surgery, yes (%)	11 (22.4)	6 (28.6)	0.58
Lung retransplantation (%)	0 (0)	1 (4.8)	

IPF: idiopathic pulmonary fibrosis; IPAH: idiopathic pulmonary arterial hypertension; HSCT: hematopoietic stem cell transplantation

**Fig. 3:**
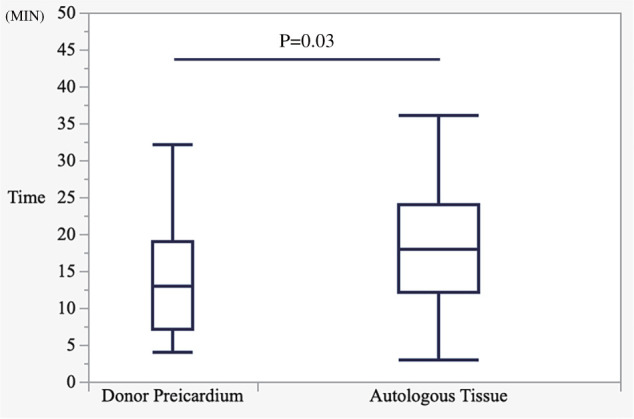
Box-and-whisker plot of pericardial closure time. Pericardial closure time was significantly shorter in the Donor pericardium group (mean: 13 min; interquartile range: 7–19) than in the Autologous tissue group (mean: 19 min; interquartile range: 12–24) (P = 0.03).

**Fig. 4:**
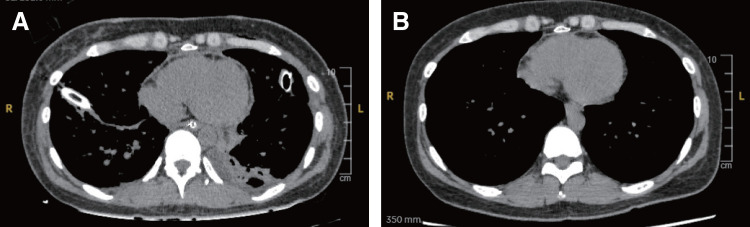
(**A**) CT scan during acute lung rejection after transplantation. (**B**) CT scan at 6 months postoperatively. CT: computed tomography

For the primary outcome, the duration of pericardial closure was significantly shorter in the Donor pericardium group (mean: 13 min, interquartile range: 7–19) than in the Autologous tissue group (mean: 19 min; interquartile range:12–24) (P = 0.03) (**Fig. 3**). For the secondary outcome, no significant differences in the incidence of heart failure, constrictive pericarditis, or pericardial effusion within 1 year postoperatively were observed between the groups (**Table 2**). Within 1 year postoperatively, heart failure occurred in 2 patients (4.1%) in the Autologous tissue group and 1 patient (4.7%) in the Donor pericardium group (P = 0.89). Pericardial effusion was observed in 5 patients (10%) in the Autologous tissue group and 2 patients (9.5%) in the Donor pericardium group (P = 0.93). Constrictive pericarditis confirmed by right heart catheterization occurred in 1 patient (2%) in the Autologous tissue group and 1 patient (4.7%) in the Donor pericardium group (P = 0.54). All cases of constrictive pericarditis were managed conservatively with medical therapy without requiring surgical pericardiectomy. Acute lung rejection occurred in 14 (20%) and 7 (10.3%) patients in the Autologous tissue and the Donor pericardium groups, respectively (P = 0.51); however, none showed evident pericardial thickening or increased pericardial effusion in the Donor pericardium group (**Figs. 4A** and **4B**). In a case of delayed chest closure performed on postoperative day 5, the donor pericardium showed no abnormalities in color or texture (**Fig. 2B**). In this study, only 1 case of lung retransplantation was performed, and in that case, autologous tissue had been used for pericardial closure during the initial surgery. At the time of retransplantation, adhesions were observed between the autologous tissue and the heart (**Video 2**).

**Table 2:** Outcomes of each pericardial closure techniques

	Autologous (n = 49)	Donor (n = 21)	*P*-value
Primary outcome			
Pericardial closure time, mean (IQR)	19 (12–24)	13 (7–19)	0.03
Secondary outcomes			
Complications			
Heart failure, n (%)	2 (4.1)	1 (4.7)	0.89
Pericardial effusion, n (%)	5 (10)	2 (9.5)	0.93
Pericarditis, n (%)	1 (2)	1 (4.7)	0.54
Acute pulmonary rejection, yes (%)	14 (20.6)	7 (10.3)	0.51

IQR: interquartile range

## Discussion

This study demonstrates that donor pericardium allows technically simpler and faster pericardial closure compared with autologous tissue in DDLT. Rather than focusing on long-term clinical outcomes, our findings highlight procedural feasibility and intraoperative simplicity without apparent short- to mid-term safety concerns. Although the absolute difference in closure time was approximately 6 min, this reduction likely reflects simplification of the procedure rather than mere time savings. Pericardial closure is performed at the end of the operation, after separation from extracorporeal support. At this stage, the heart often remains enlarged, and wider coverage of the pericardial defect may be required. Donor pericardium allows broader coverage of the pericardial defect and can be easily tailored by simple trimming to accommodate large or irregular defects. In contrast, the amount of available autologous tissue varies among patients and may be insufficient to cover a large defect adequately. In addition, autologous tissues often require trimming and adjustment before closure. Minimizing these additional steps may contribute to smoother operative workflow and improved reproducibility. Moreover, preparation of autologous tissues, including trimming and shaping, was included in the closure time, suggesting that the observed difference represents procedural streamlining inherent to the donor pericardium technique. In contrast, preparation of donor pericardium is typically performed on the back table during donor lung preparation by the back-table team and is therefore not included in the pericardial closure time measured at the operative field.

The use of pericardium in transplantation is diverse, regardless of whether it is donor-derived or autologous. We have previously reported the use of donor pericardium to correct discrepancies in pulmonary artery size,^2)^ as well as the use of an autologous pericardial conduit between the recipient’s upper pulmonary vein and the donor’s lower pulmonary vein to perform pulmonary venoplasty in living-donor lung transplantation.^3)^ Casula et al. has also reported the application of donor pericardium in DDLT to correct discrepancies in the left atrial cuff.^4)^ Beyond lung transplantation, donor pericardium has been used for repair in cases of traumatic globe rupture.^5)^ Therefore, the use of donor pericardium for pericardial closure can be considered as one of the effective ways of fulfilling the functional role of the pericardium.

Reportedly, postoperative constrictive pericarditis following lung transplantation has an incidence of 0.4%–0.8%.^6–8)^ Proposed causes include pericardial manipulation, as in bilateral lung transplantation,^6–10)^ underlying diseases such as idiopathic pulmonary fibrosis^7)^ or lymphangioleiomyomatosis,^9)^ previous episodes of acute cellular rejection,^10)^ postoperative cytomegalovirus infection,^9)^ and the use of antifungal agents.^8)^ However, the precise cause remains unclear, and its association with pericardial closure has not been discussed. The incidence of pericarditis reported in this study was slightly higher than in the literature;^6–8)^ however, none of the cases required surgical intervention. Additionally, acute lung rejection occurred but did not have an impact on the donor pericardium clinically. In addition, limited macroscopic observation during delayed chest closure revealed no apparent abnormalities in the donor pericardium in the early postoperative period.

Beyond short-term safety considerations, the potential impact of pericardial reconstruction on future reentry warrants attention. Pericardial reconstruction may also have implications for adhesion formation in cases requiring reentry. In cardiac surgery, the use of adhesion barriers or pericardial substitutes has been reported to facilitate safer reoperations by reducing dense adhesions between the sternum and heart.^11)^ Although the materials used in those reports differ from donor pericardium, these findings support the concept that restoration of a physical barrier over the heart may mitigate adhesion-related risks during reentry. In the present study, adhesions between the autologous tissue and the heart were observed at the time of retransplantation in 1 case. While no retransplantation cases were available in the Donor pericardium group, biologic reconstruction using donor pericardium restores the pericardial layer itself and may therefore theoretically provide a similar barrier effect. However, this possibility remains speculative and warrants further investigation with longer follow-up and additional reoperative cases.

This study has several limitations. First, we acknowledge the potential for era-related bias, as autologous tissue closure was performed in earlier years and donor pericardium was introduced in 2020. Improvements in operative efficiency over time or accumulation of team experience may have influenced the observed difference in closure time. However, the core surgical team, operative workflow, and perioperative management strategies remained largely consistent throughout the study period. The introduction of donor pericardium represented a change in closure material rather than a fundamental modification of the operative procedure. Nevertheless, the potential impact of temporal factors cannot be completely excluded and should be considered when interpreting the results. Second, it only presents short- to mid-term outcomes. In the long term, as more cases accumulate, it will be necessary to evaluate clinical outcomes, including adverse events and assessments during lung retransplantation. Notably, there was only 1 case of lung retransplantation in this study, and no retransplantation cases have yet been reported among patients in whom donor pericardium was used. In the case where autologous tissue was used for pericardial closure during the initial surgery, adhesions were observed between the autologous tissue and the heart at the time of retransplantation. Whether the use of donor pericardium can reduce adhesions during lung retransplantation remains unknown. Third, although the potential impact of rejection against donor pericardium remains unclear, this study was not designed to evaluate immunological responses specific to donor pericardium.

## Conclusion

Pericardial closure using donor pericardium is a technically simple approach that enables faster closure without obvious short-term adverse signals.

## Supplementary Materials

Video 1: First, when using autologous tissues, the thymus is trimmed and sutured to the autologous pericardium. Second, when using the donor’s pericardium, the donor pericardium is used to cover the defect in the autologous pericardium and sutured closed.

Video 2: During lung retransplantation in a patient whose pericardium had been closed with autologous tissue at the initial surgery, adhesions were present between the autologous tissue used for pericardial closure and the heart.

## Declarations

### Data availability

The data underlying this article will be shared on reasonable request to the corresponding author.
